# Global trends in lumbar spondylolysis research: A comprehensive bibliometric analysis

**DOI:** 10.1097/MD.0000000000049371

**Published:** 2026-06-19

**Authors:** Yan Wang, Xiaojing Guo, Yan Wang, Chunsheng Qian

**Affiliations:** a Department of Rehabilitation Medicine, The 962nd Hospital of the Chinese People’s Liberation Army Joint Logistics Support Force, Harbin, Heilongjiang, China; b Second Clinical Medical College, Shanxi University of Traditional Chinese Medicine, Jinzhong, Shanxi, China; c Rehabilitation Center, The Second Affiliated Hospital of Heilongjiang University of Chinese Medicine, Harbin, Heilongjiang, China.

**Keywords:** bibliometric analysis, Citespace, lumbar Spondylolysis, VOSviewer

## Abstract

**Objective:**

This study conducted a visual analysis of literature related to lumbar spondylolysis from January 2015 to August 2025, summarizing the current state, research hotspots, and developing trends in this field to provide theoretical support for subsequent research and clinical practice.

**Methods:**

The China National Knowledge Infrastructure and Web of Science databases were searched. Using CiteSpace V6.3.R1 and VOSviewer 1.6.20 (Leiden University ), visualization maps were generated and analyzed from perspectives including publication volume, authors, institutions, cited journals, and keywords.

**Results:**

The search identified 602 articles from Web of Science and 268 from China National Knowledge Infrastructure. Analysis of both databases revealed distinct research networks, centered on high-output authors and collaborative institutions. The leading research institutions were primarily hospitals and clinics, with Harvard Medical School being the most prolific. Among authors, Sairyo, Koichi had the highest number of publications and citations, while Spine was the most cited journal. Early research, both in Chinese and international literature, primarily addressed surgical interventions and imaging studies. Chinese core journals frequently emphasized “internal fixation” and adolescent populations, whereas international studies focused more on “low back pain” and diagnostic “tomography.” In recent years, conservative treatment, risk factors, and rehabilitation management have gained prominence, marking a shift in research emphasis from surgical intervention toward disease prevention and rehabilitation.

**Conclusion:**

Research on lumbar spondylolysis is increasingly characterized by multimodality and multidisciplinary integration, with a growing convergence in research priorities within the global academic community. However, China continues to trail behind leading international efforts in this domain. Moving forward, priorities should include conducting high-quality prospective cohort studies, multicenter comparative trials, and multimodal cross-disciplinary research to enhance evidence-based clinical decision-making and advance personalized rehabilitation management.

## 1. Introduction

Lumbar spondylolysis is characterized by a bony defect or fracture in the pars interarticularis of the vertebral arch, most frequently occurring at the L5 level. It commonly results from repetitive extension-rotation stress and is often observed in adolescents, athletes, military personnel, and other populations subjected to recurrent back loading.^[1,2]^ Affected patients typically present with recurrent low back pain. In severe cases, the condition may lead to lumbar instability or spondylolisthesis, ultimately restricting mobility and impairing both quality of life and athletic performance.^[3]^ With the aging of the general population, the growth of competitive sports and national fitness initiatives, and continuous advancements in imaging technology, the clinical detection rate of lumbar spondylolysis has been increasing.^[4]^ Consequently, achieving early accurate diagnosis and effective intervention has become a major research priority and a clinical challenge in this field.

Although research output has grown in recent years, several critical gaps remain. First, despite the expanding volume of literature, systematic integration of existing evidence is limited, and research hotspots and evolutionary trends are not well delineated. Second, most existing reviews rely on manual literature screening and synthesis, which introduces considerable subjectivity and limits the ability to reveal underlying knowledge structures and latent thematic connections. Third, there is a lack of systematic synthesis of clinical evidence-based findings, which has impeded the establishment of standardized diagnostic and therapeutic pathways.

Therefore, there is an urgent need for a more scientific, objective, and systematic methodology to integrate and analyze the body of research on lumbar spondylolysis. This study utilizes CiteSpace V6.3.R1 (Drexel University) to perform a visual analysis of domestic and international literature related to lumbar spondylolysis. Through this approach, we aim to systematically outline the current research landscape, trace the discipline’s developmental trajectory, and identify emerging frontiers, thereby providing new insights to support clinical decision-making and guide future academic research.

## 2. Data and methods

### 2.1. Data source

The search period was set from January 2015 to August 2025. For Chinese databases, advanced search methods were employed using the following strategy: subject terms = (lumbar pars interarticularis spondylolysis OR lumbar pars interarticularis defect OR pars interarticularis spondylolysis OR pars interarticularis defect). For the Web of Science (WOS) database, the WOS Core Collection was selected with the index set to “SCI-EXPANDED.” The specific search strategy was TS = (spondylolysis OR “pars interarticularis defect*” OR “lumbar spondylolysis”). The search was completed and data exported on August 19, 2025.

### 2.2. Inclusion and exclusion criteria

Inclusion Criteria: Chinese language for China National Knowledge Infrastructure (CNKI) database; English language for WOS database; CNKI: “Academic Journal” type, WOS: “Article OR Review Article”; all literature relevant to search terms with full-text availability.

Exclusion Criteria: CNKI: degree theses, conferences, newspapers; WOS: editorial material, letter,meeting abstract, early access, proceeding paper, correction, reprint; duplicate publications and articles with incomplete information.

### 2.3. Research methodology

**Figure 1.**
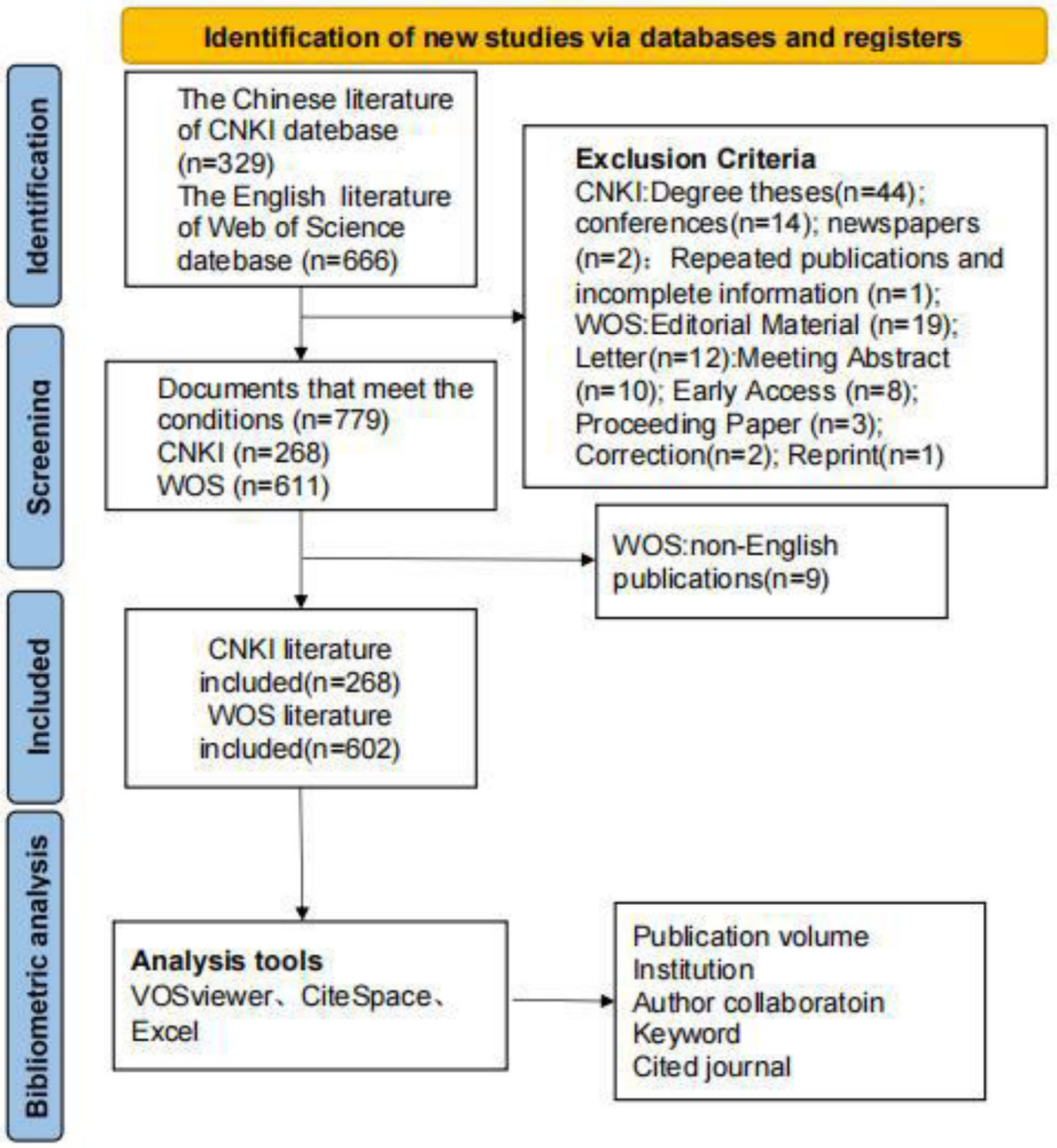
Flow chart of literature selection.

The retrieved Chinese and English literature was saved under the filename format “download_x-x.txt” and processed using CiteSpace 6.2.R6 for deduplication. Ultimately, 602 publications from the WOS database and 268 from the CNKI database were included. In CiteSpace, the time slicing was set from 2015 to 2025, with years per slice of “1.” The data were converted into a compatible format using the built-in CiteSpace converter. Visual analyses were subsequently performed based on publication volume, institutions, authors, cited journals, as well as keyword co-occurrence, clustering, and burst detection (Fig. 1).

## 3. Results

### 3.1. Visual analysis of publication volume

**Figure 2.**
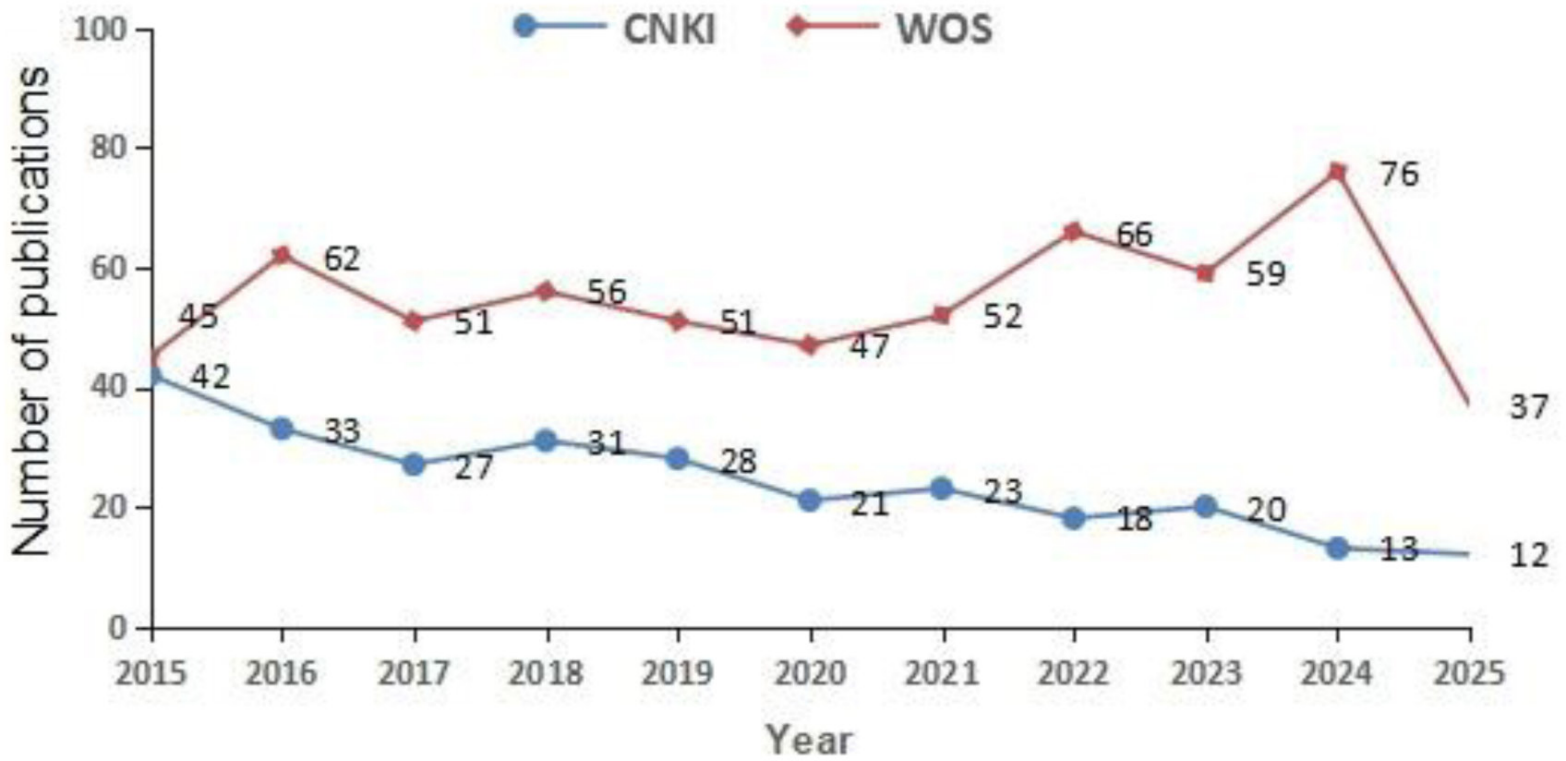
The number of publications on lumbar spondylolysis from 2015 to 2025.

The annual publication output of the included literature is summarized in Figure 2. Over the past decade, a total of 602 English-language articles were published, demonstrating an overall fluctuating but increasing trend, with a peak of 76 publications in 2024. In contrast, 286 Chinese-language articles were published during the same period, showing a general decline from 2015 to 2025. These trends suggest that while lumbar spondylolysis remains an active research area globally, the volume of research output in China has experienced a moderate decrease in recent years.

### 3.2. Visualization of institutional analysis

**Figure 3.**
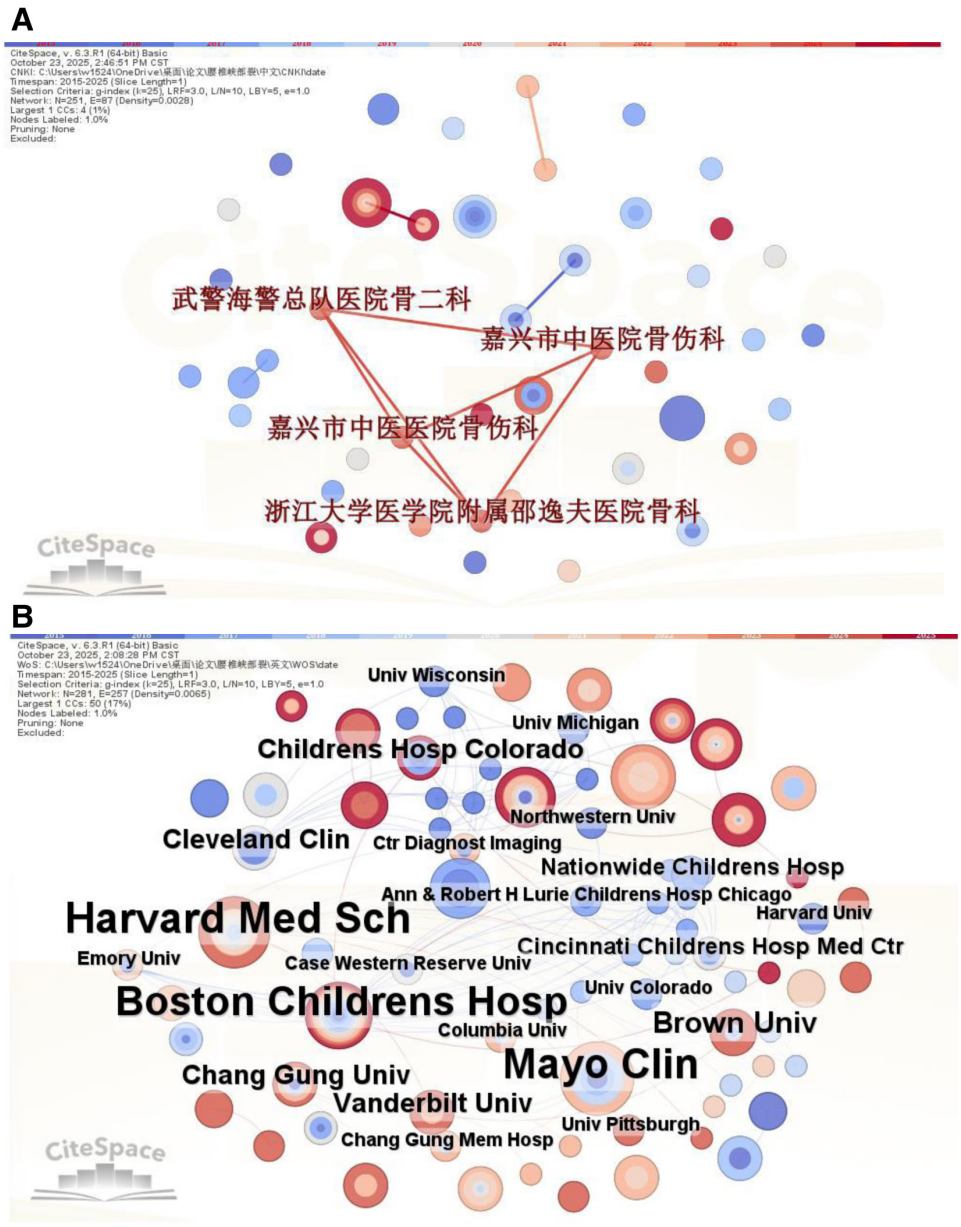
Co-occurrence map of institutional collaboration networks in (A) the CNKI database; (B) and the WOS database. Each circle represents an institution, with circle size proportional to publication volume. Purple circles’ size correlates with centrality, while line thickness indicates the strength of connections between institutions. CNKI = China National Knowledge Infrastructure, WOS = Web of Science.

Analysis based on Chinese databases identified 251 institutions that have published articles in this field, with hospitals demonstrating the highest research output. The top 3 institutions by publication volume were the Department of Spine Surgery, First Affiliated Hospital of Guangxi University of Chinese Medicine (5 articles); the Department of Spinal Orthopedics, Nanfang Hospital, Southern Medical University (4 articles); and the Department of Spine Surgery, PLA 188th Hospital (4 articles).In WOS, 257 institutions published relevant literature, among which 3 institutions produced ≥ 10 articles: Harvard Medical School (12 articles), Mayo Clinic (11 articles), and Boston Children’s Hospital (10 articles). Research output from Harvard Medical School has been concentrated particularly in the period since 2019. Collaboration network analysis conducted with CiteSpace revealed 251 nodes and 87 links in the Chinese database, with a network density of 0.0028. Limited collaborative linkages were observed among a small number of institutions, including the Second Department of Orthopedics of the Armed Police Coast Guard General Hospital, the Orthopedics Department of Jiaxing Traditional Chinese Medicine Hospital, the Orthopedics Department of Jiaxing Hospital of Traditional Chinese Medicine, and the Orthopedics Department of Sir Run Run Shaw Hospital, Zhejiang University School of Medicine. Overall, institutional connections were sparse, with minimal cross-provincial or interdisciplinary collaboration. In contrast, the WOS-based network contained 281 nodes and 257 links, resulting in a network density of 0.0065. This indicates that research collaborations are largely concentrated among United States institutions, although inter-institutional cohesion remains relatively weak (Figs. 3A and 3B). Centrality values for all institutions in both databases were low (< 0.03), suggesting that no single institution has yet attained a dominant influential role in this research domain.

### 3.3. Visualization of author collaboration analysis

**Figure 4.**
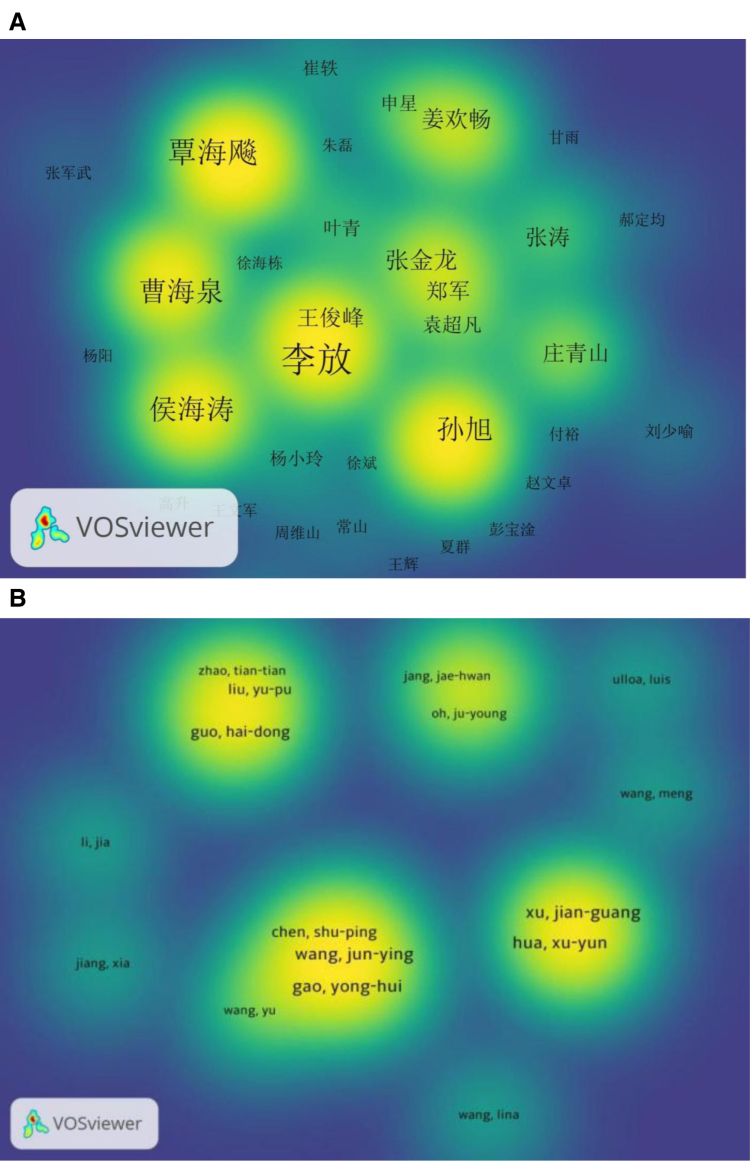
Density map of the author collaboration network in (A) the CNKI database; (B) and the WOS database. In the network map, higher author density corresponds to colors closer to bright yellow, while lower density approaches blue. Density values depend on the strength of author connections and their importance within the surrounding regions. CNKI = China National Knowledge Infrastructure, WOS = Web of Science.

In the Chinese database, 6 authors have published 5 or more articles: Li Fang (李放), Zhang Zhicheng (张志成), Zhang Tao (张涛), Li Songkai (李松凯), Qin Haibiao (覃海飚), and Chen Yongxi (陈勇喜). Among them, Li Fang ranks first in both publication count (8) and total link strength (31). According to Price Law, the formula for determining core authors is M = 0.749 × √Nmax, where Nmax represents the highest number of publications by a single author. With Nmax = 8, M ≈ 2.12; thus, authors with at least 2 publications were defined as core authors, resulting in 113 individuals in this field. A coauthorship density map was generated using VOSviewer (Leiden University) to visualize collaborative relationships and research intensity among influential authors in key areas, with weights set to total link strength (Fig. 4A). Among the 286 Chinese publications, a total of 1037 authors were involved. The network diagram displays only authors with 2 or more publications, revealing 30 core research teams (including those led by Ma Tieming, Tang Chenglin, and Fang Jianqiao) with the largest team consisting of 12 members. For clarity, not all team members are displayed. In the WOS database, the most prolific author has published 17 papers, and 7 authors have published 10 or more articles (Table 1). Applying Price Law to the 2944 authors in WOS (M ≈ 3), and setting a minimum threshold of 3 publications, 121 core authors were identified (Fig. 4B). These authors primarily form research teams centered around Aoki, Yasuchika; Yamazaki, Masashi; Higashino, Kosaku; and Kakutani, Kenichiro. These teams generally consist of researchers from the same university and its affiliated hospitals, or from the same geographic region.

**Table 1:** Top 7 institutions by publication volume in the WOS database.

Author	Documents	Citations	Total link strength
Sairyo, Koichi	17	181	78
Sakai, Toshinori	13	148	65
Higashino, Kosaku	11	141	57
Tezuka, Fumitake	11	116	56
Yamazaki, Masaki	11	68	56
Takata, Yoichiro	10	61	54
Tatsumura, Masaki	10	132	53

WOS = Web of Science.

### 3.4. Keywords

#### 3.4.1. Keywords co-occurrence visualization analysis

**Figure 5.**
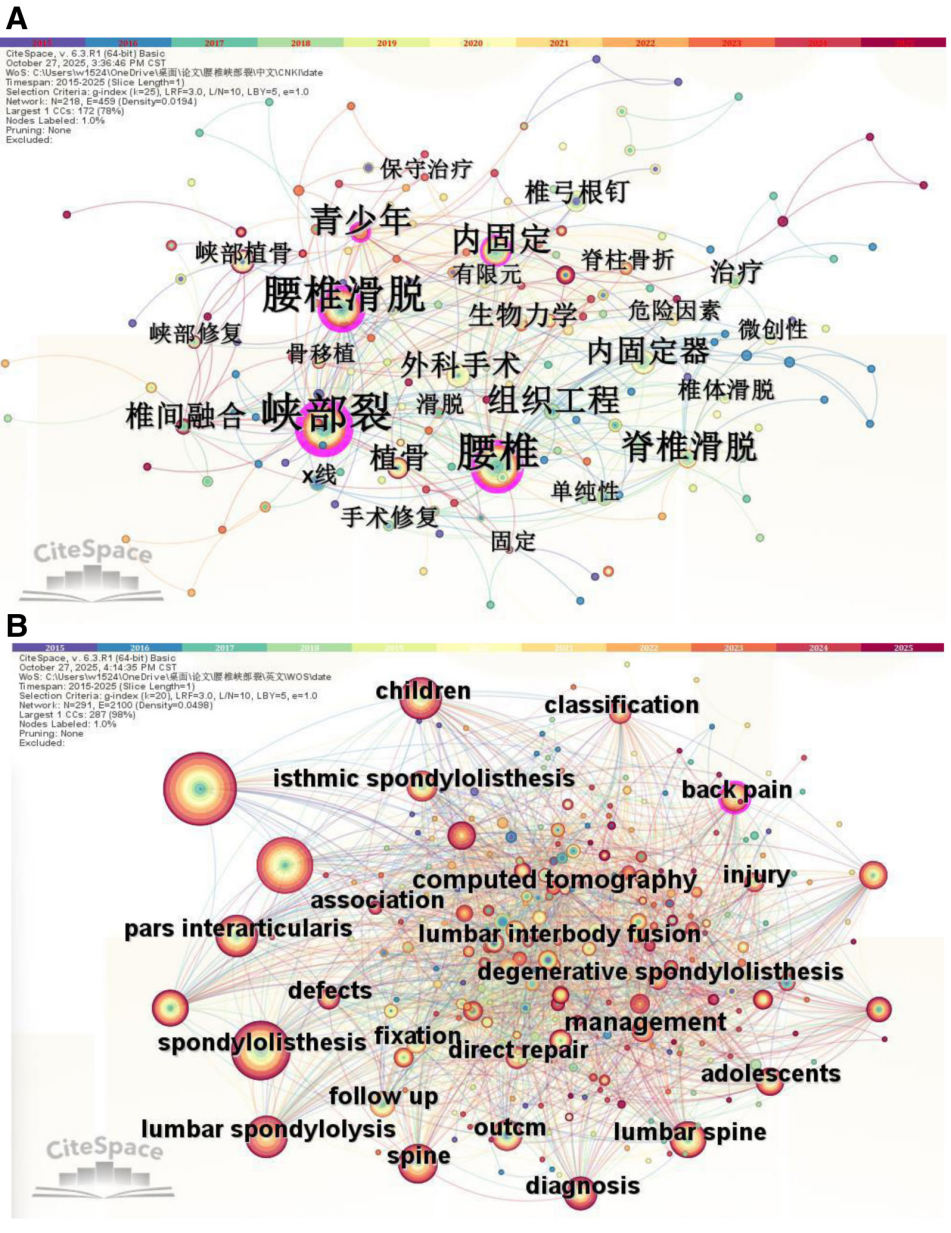
Co-occurrence network diagram of keywords in (A) the CNKI database; (B) and the WOS database. CNKI = China National Knowledge Infrastructure, WOS = Web of Science.

In bibliometric analysis, the frequency of keyword co-occurrence can reveal research hotspots and emerging trends within a given field.^[5]^ Using CiteSpace, we extracted 218 keywords from CNKI and 291 from WOS. To focus on content-related themes rather than search-related terms, we excluded generic and query-specific phrases such as “isthmic fracture (峡部裂) ,” “lumbar spine (腰椎) ,” “lumbar spondylolisthesis (腰椎滑脱) ,” “spinal spondylolisthesis (脊椎滑脱) ,” “spondylolysis,” “spondylolisthesis,” “lumbar spondylolysis,” and other terms directly tied to the search strategy. The top 10 keywords from each database are listed in Tables 2 and 3. In the Chinese database, “internal fixation” (内固定) appeared most frequently, while “adolescents” (青少年) showed the highest centrality. In the WOS database, “low back pain” was the most frequent keyword (159 occurrences), and “computed tomography” had the highest centrality (0.1), as visualized in Figures 5A and 5B.

**Table 2:** Keyword frequency and centrality in the CNKI database.

Rank	Keyword	Frequency	Keyword	Centrality
1	Internal fixation (内固定)	18	Internal fixation(内固定)	0.18
2	Adolescents (青少年)	14	Adolescents (青少年)	0.15
3	X-ray radiograph (x线平片)	12	Spinal Fusion (椎间融合)	0.09
4	Internal fixation device (内固定器)	9	Physical Examination (体格检查)	0.08
5	Surgical procedure (外科手术)	9	Isthmic bone grafting (峡部植骨)	0.05
6	Isthmic bone grafting (峡部植骨)	9	Pedicle screw (椎弓根钉)	0.05
7	Pedicle screw (椎弓根钉)	8	Lumbar Pain (腰痛)	0.05
8	Bone grafting (植骨)	7	Evidence-Based Research (实证研究)	0.05
9	Spinal fusion (椎间融合)	6	Bone Grafting (植骨)	0.04
10	Tissue engineering (组织工程)	6	X-ray radiograph (x线)	0.04

CNKI = China National Knowledge Infrastructure.

**Table 3:** WOS keyword frequency and centrality.

Rank	Keyword	Frequency	Keyword	Centrality
1	low back pain	159	Computed tomography	0.10
2	children	116	Management	0.09
3	spine	13	Association	0.09
4	pars interarticularis	99	Diagnosis	0.08
5	lumbar spine	88	Classification	0.08
6	natural history	79	Injury	0.08
7	prevalence	69	Spine	0.07
8	Diagnosis	62	Prevalence	0.07
9	adolescents	61	Defects	0.07
10	management	51	direct repair	0.06

WOS = Web of Science.

#### 3.4.2. Visualization of keyword cluster analysis

**Figure 6.**
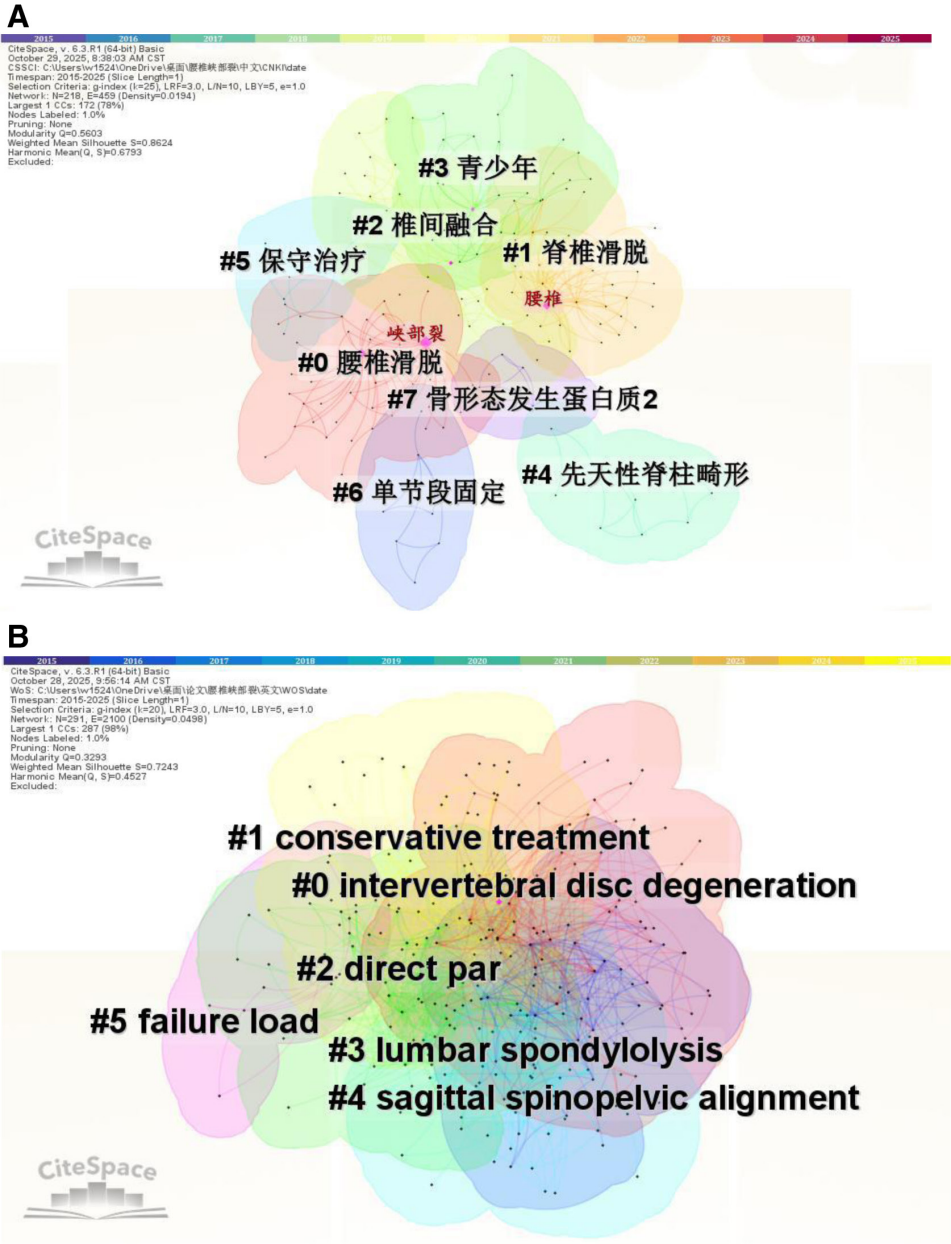
Keyword clustering diagram from (A) the CNKI database; (B) and the WOS database. CNKI = China National Knowledge Infrastructure, WOS = Web of Science.

Keyword clustering analysis was conducted using CiteSpace to generate keyword cluster maps (Figs. 6A and 6B). Based on the log-likelihood ratio algorithm, 11 Chinese and 6 English keyword clusters were identified, as summarized in Tables 4 and 5. According to established criteria, a silhouette value (S) > 0.7 indicates convincing cluster homogeneity, and a modularity value (Q) > 0.3 reflects significant clustering structure.^[6]^ The Chinese keyword network yielded S = 0.8624 and Q = 0.5603, while the WOS network produced S = 0.7243 and Q = 0.3293, demonstrating robust cluster homogeneity and structurally meaningful mapping. Among the Chinese keyword clusters, labels #0 and #1 represent the largest groups, underscoring the central role of diagnosis and treatment in lumbar spondylolysis research. Clusters #2, #3, #5, #7, and #9 highlight adolescents, military personnel, pilots, and obese individuals as high-risk populations. Clusters #4, #9, and #10 exhibited high internal coherence, suggesting emerging or specialized research directions. In the WOS database, keyword clusters were labeled as follows: #0 intervertebral disc degeneration, #1 conservative treatment, #2 direct pars repair, #3 lumbar spondylolysis, #4 sagittal spinopelvic alignment, and #5 failure load. Clusters #0 and #1 were the largest in scale; however, cluster #1 demonstrated substantially higher quality than #0, reflecting a more focused and mature research stream in conservative treatment. Cluster #5, though the smallest (8 nodes), achieved the highest silhouette value (0.907), indicating strong internal consistency. Overall, research hotspots concentrate on disease pathogenesis, clinical diagnosis, biomechanical assessment, and treatment modalities, reflecting a multidimensional research landscape in this field.

**Table 4:** Keyword clustering information table from the CNKI database.

ClusterID	Size	Silhouette	(LLR) Label
#0	43	0.743	Lumbar spondylolisthesis; Isthmus fracture; Pedicle fracture; Plain radiograph; CT diagnosis
#1	36	0.813	Spinal spondylolisthesis; Lumbar spondylolisthesis; Tissue engineering; Lumbar spine; Spinal implants
#2	29	0.961	Intervertebral fusion; Young soldiers; Pedicle fracture; Spondylolisthesis; Repair
#3	25	0.858	Adolescents; Spinal fractures; Review articles; Spinal spondylolisthesis; Lumbar spondylolisthesis
#4	9	0.979	Congenital spinal deformities; Flight trainees; Flight personnel; Classification manifestations; Guidelines
#5	7	0.937	Conservative treatment; Direct repair; Bone healing; Youth population; Risk factors
#6	7	0.972	Single-segment fixation; Isthmus repair; Nonfusion; Pedicle screws; Bone-inductive active materials
#7	6	0.942	Bone morphogenetic proteins; Transplantation; Autologous; Screw-hook internal fixation; Young adults
#8	4	0.987	Dynamic health education; Complications; Targeted management; Rehabilitation; Lumbar spondylolysis
#9	3	0.996	Minimally invasive; Obesity; Cortical bone canal; Lumbar degenerative disease; Pedicle screws
#10	3	1	Lumbar function; Minimally invasive transforaminal lumbar interbody fusion; Quadrant canal; Pain intensity; Open transforaminal interbody fusion

CNKI = China National Knowledge Infrastructure, LLR = log-likelihood ratio.

**Table 5:** Keyword clustering information from the WOS database.

ClusterID	Size	Silhouette	(LLR) Label
#0	63	0.622	intervertebral disc degeneration; lumbar spine; male pole vaulter; low back pain; descriptive analysis
#1	63	0.751	conservative treatment; adolescent athlete; adolescent lumbar spondylolysis; pediatric lumbar spondylolysis; early-stage lumbar spondylolysis
#2	59	0.759	direct par; young patient; pars defect; literature review; lumbar spondylolysis
#3	54	0.727	Lumbar spondylolysis; degenerative spondylolisthesis; lumbar spondylolisthesis; interbody fusion; high-grade spondylolisthesis
#4	40	0.751	sagittal spinopelvic alignment; sacral slope; pediatric spine pathologies; multisegmental spondylolysis; low lumbar curvature
#5	8	0.907	failure load; facet deflection stiffness strain; quantitative evaluation; spinal kinematics; ground reaction force

Cluster numbers are inversely proportional to cluster size. For example, #0 represents the largest cluster, and so on.

LLR = log-likelihood ratio, WOS = Web of Science.

#### 3.4.3. Keyword with the strongest citation bursts visualization

**Figure 7.**
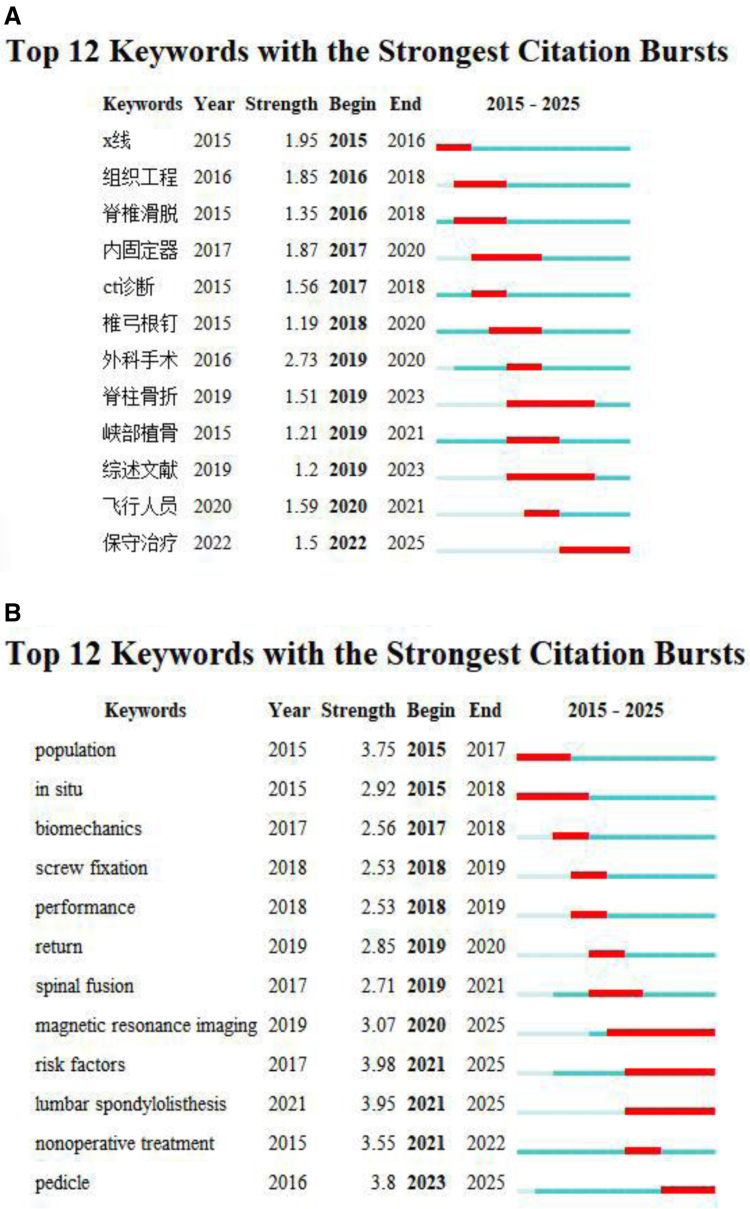
Keywords with the strongest citation bursts from (A) the CNKI database; (B) and the WOS database. CNKI = China National Knowledge Infrastructure, WOS = Web of Science.

Burst analysis was performed using CiteSpace to identify evolving research trends and emerging topics in lumbar spondylolysis.^[7]^ Figures 7A and 7B present the top 12 keywords with the strongest citation bursts in published studies. The blue line represents the time interval, and the red line indicates the burst duration. Overall, burst strength in both Chinese and English databases was relatively low, with short durations, suggesting rapidly shifting research focuses in this field. In the Chinese database, research hotspots evolved through distinct phases: from 2015 to 2018, burst keywords such as “tissue engineering (组织工程),” “internal fixation devices (内固定器),” and “CT diagnosis (CT诊断),” indicating an early focus on fundamental materials science and imaging techniques; from 2019 to 2021, keywords like “pedicle screws (椎弓根钉),” “surgical procedures (外科手术) ,” and “spinal fractures (脊柱骨折) ” appeared frequently, reflecting a shift in research focus toward surgical interventions and repair of spinal structural defects. Since 2022, “conservative treatment (保守治疗)” has emerged as a prominent theme, signaling growing interest in nonsurgical and rehabilitation-based management, and marking a diversification of treatment strategies. In the WOS database, the evolution of keywords revealed a more systematic progression: during 2015 to 2018, terms such as “in situ” and “biomechanics” highlighted foundational work on biomechanical mechanisms and local anatomy. From 2018 to 2020, keywords like “screw fixation,” “performance,” and “return” indicated a focus on fixation techniques, surgical outcomes, and postoperative functional recovery. Since 2021, international research has shifted toward epidemiological risk factors, degenerative disease management, and nonsurgical treatment options. These trends illustrate that while surgical intervention and internal fixation have long dominated the global research agenda, recent years have witnessed a growing emphasis on integrating conservative management and rehabilitation: a shift that may define future research directions.

#### 3.4.4. Visual analysis of cited journals

**Figure 8.**
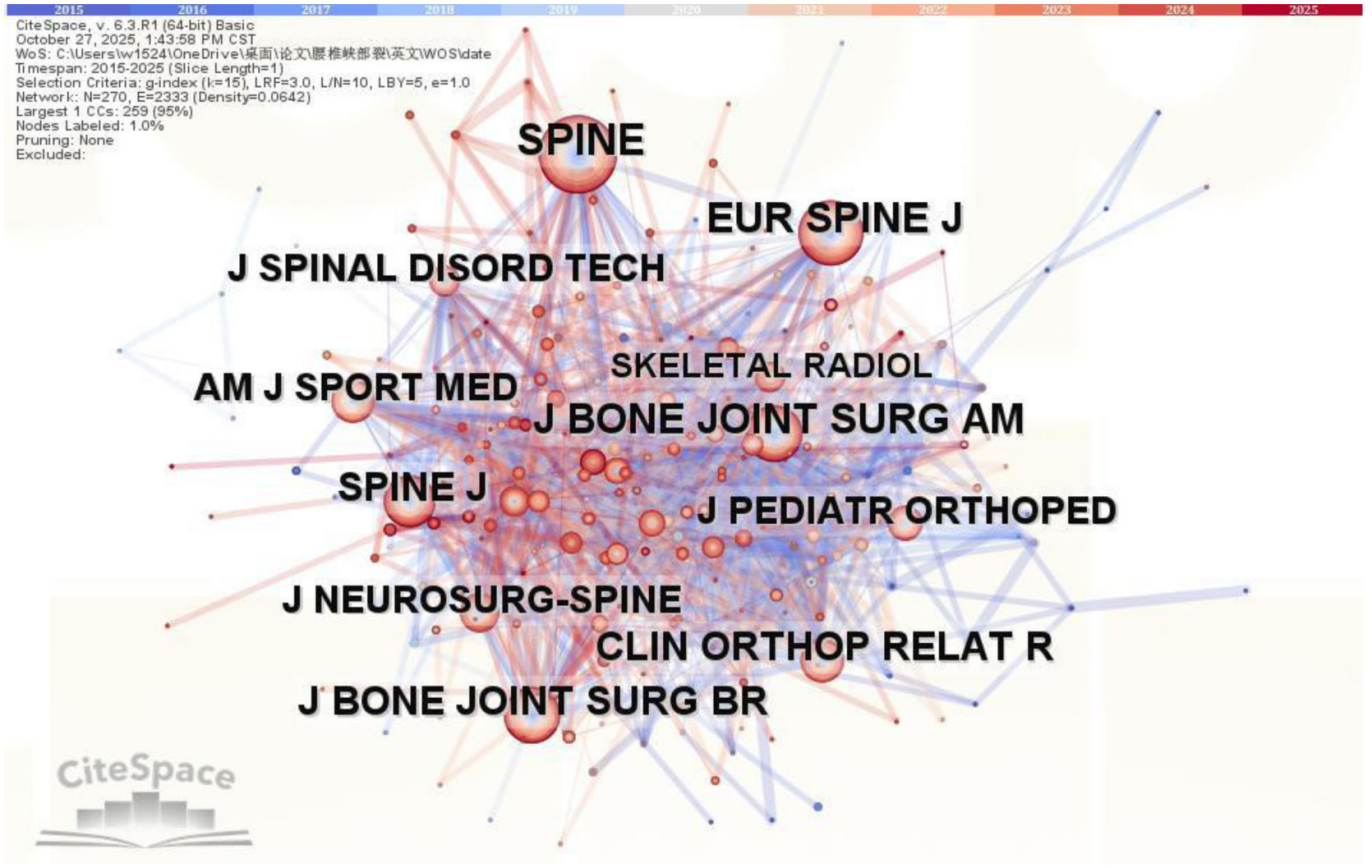
Visual analysis map of WOS-cited journals. WOS = Web of Science.

The frequency of journal citations reflects their academic influence in the field of lumbar spondylolysis research. Due to technical limitations of bibliometric software, visualization analysis could only be performed for journals indexed in the WOS database. Between 2015 and 2025, a total of 270 distinct journals published relevant literature, forming a network with 2333 links and a density of 0.0642. The journals exhibited a well-connected network, with 11 journals each receiving more than 170 citations (Table 6 and Fig. 8). The journal Spine had the highest citation count (549 citations) and the highest centrality (0.1). The average impact factor of the top 11 cited journals was 3.37. Among these, journals such as Spine J, Am J Sport Med, J Bone Joint Surg Am, and Clin Orthop Relat R demonstrated high impact factors and top-tier rankings in their categories, confirming their established authority in this research domain.

**Table 6:** The top 11 cited journals in WOS.

Journal	Frequency	2025-IF	JCR
Spine	549	3.5	Q1
Eur Spine J	430	2.7	Q2
J Bone Joint Surg Am	363	4.3	Q1
J Bone Joint Surg Br	309	4.4	Q1
Spine J	291	4.7	Q1
Clin Orthop Relat R	267	4.4	Q1
Am J Sport Med	223	4.5	Q1
J Neurosurg Spine	207	3.1	Q
J Pediatr Orthoped	178	1.5	Q3
J Spinal Disord Tech	176	2.3	Na
Skeletal Radiol	171	2.2	Q2

IF = impact factor, JCR = journal citation reports, WOS = Web of Science.

## 4. Discussion

### 4.1. Research status

Over the past decade, research on lumbar spondylolysis has remained highly prominent in the global academic community. The observed decline in publications indexed in the WOS database in 2025 may be attributed to the retrieval cutoff date of August 2025, resulting in an incomplete dataset. Nevertheless, the overall trend continues to reflect the expanding depth and scope of international research. In contrast, the volume of publications in China’s CNKI database has shown a steady decline since 2015, suggesting insufficient momentum in domestic research efforts. This disparity underscores the need for Chinese scholars to further focus on key scientific issues in both basic and clinical research to enhance the quality and originality of their outputs.

Institutional analysis within the CNKI database reveals that clinical hospitals constitute the main research entities, with the Spine Surgery Department of the First Affiliated Hospital of Guangxi University of Chinese Medicine ranking first (5 publications). Collaborative efforts are largely confined to individual hospitals or within provincial boundaries, with limited cross-regional cooperation. All institutions exhibit low centrality values (< 0.03), indicating the absence of a dominant research hub. In comparison, internationally renowned institutions such as Harvard Medical School and the Mayo Clinic lead the field, supported by a significantly higher collaboration network density than their Chinese counterparts. However, overall network cohesion remains suboptimal, with partnerships often restricted to local or institutional boundaries. Therefore, fostering cross-institutional collaboration and promoting synergistic innovation among hospitals, universities, and research institutes is essential.

In terms of author influence, Li Fang emerged as the most prolific researcher in the Chinese database, contributing to the formation of 30 core research teams: the largest of which comprised 12 members. On the international stage, Sairyo Koichi stands as a leading authority, having established a stable research consortium centered around Japanese scholars Aoki and Yamazaki, characterized by high overall connection strength. These findings illuminate the current state and challenges in institutional and author-level collaboration, offering valuable insights for future research planning and partnership strategies.

Journal co-citation analysis indicates that studies on lumbar spondylolysis are most frequently published in Spine (Impact Factor = 3.5, Q1), the leading journal in the field. Among the top 11 co-cited journals, the majority belong to the journal citation reports Q1 quartile, with an overall network density of 0.0642, reflecting strong inter-journal connectivity. Academic Emergency Medicine recorded the highest centrality (0.1), serving as a key bridge in interdisciplinary knowledge exchange. This pattern highlights the dual character of lumbar spondylolysis research: it maintains both specialized depth and active cross-disciplinary integration.

### 4.2. Analysis of research hotspots

#### 4.2.1. Assessment methods for lumbar spondylolysis

Accurate assessment of lumbar spondylolysis is essential for early diagnosis and timely intervention, ideally achieved through a combination of biomechanical and radiological evaluation^.[8–10]^ Domestic studies predominantly employ conventional imaging techniques such as plain radiography and computed tomography (CT), while international research places stronger emphasis on tomography (centrality = 0.1) and biomechanical parameters such as sagittal spinopelvic alignment, resulting in more systematic evaluation frameworks. Conventional imaging modalities (including radiography, CT, and magnetic resonance imaging) remain the cornerstone of clinical assessment. CT is regarded as the gold standard for diagnosis due to its high-resolution visualization of the pars defect, a status reflected by its high centrality in CiteSpace analyses.^[11]^ Magnetic resonance imaging offers distinct advantages in detecting early stress reactions, bone marrow edema, and associated disc degeneration.^[3]^ More recent investigations have increasingly incorporated 3-dimensional reconstruction and kinematic studies to better characterize dynamic changes in lumbar structure and function.^[12]^ Physical examination and functional assessment further complement imaging findings, supporting clinical screening and rehabilitation monitoring. Overall, the evaluation of lumbar spondylolysis is evolving from a single-modality imaging approach toward an integrated, multimodal strategy that emphasizes the combination of functional, dynamic, and individualized parameters; ultimately aiming to establish a dual evaluation system grounded in both biomechanical and imaging evidence.

#### 4.2.2. Research mechanisms of lumbar isthmus fractures

The pathogenesis of lumbar spondylolysis has long been a subject of investigation. Keyword clustering results indicate strong associations between pars defects and disc degeneration, abnormal sagittal spinopelvic alignment, and altered stress distribution.^[13,14]^ Existing studies confirm that the local mechanical disruption caused by spondylolysis accelerates degenerative changes in adjacent intervertebral discs, thereby exacerbating spinal instability.^[15]^ A retrospective study reported that among adult patients with spondylolytic spondylolisthesis, 33.9% exhibited significant lumbar instability classified as dynamic slippage. These patients typically presented with higher slip percentages, potentially associated with increased pelvic tilt and greater displacement of the L5 vertebra.^[16]^ Sagittal spinopelvic imbalance is regarded as a key factor contributing to stress concentration at the pars, influencing surgical outcomes, and affecting long-term prognosis.^[17]^ While international research is increasingly directed toward epidemiological risk factors, natural disease history, and long-term outcomes, domestic studies remain predominantly focused on imaging characteristics and surgical mechanisms. Overall, the field is evolving from purely anatomical explanations toward integrated analyses incorporating molecular biology, biomechanics, and systemic risk factors. This shift provides a theoretical foundation for targeted prevention and personalized rehabilitation strategies.

#### 4.2.3. Treatment strategies for lumbar spondylolysis

Our findings indicate that treatment strategies for lumbar spondylolysis are evolving from a predominantly surgical model toward an integrated and multidimensional approach. Early research primarily emphasized surgical repair and the use of internal fixation devices, particularly in adolescent populations, where surgery has been widely considered essential for restoring spinal stability and preventing disease progression.^[18,19]^ Domestic research hotspots have consistently centered on keywords such as “internal fixation” and “adolescents.” Cluster analysis further delineated a clinical pathway spanning diagnosis (#0), conservative treatment (#5), surgical intervention (#2/#6), and rehabilitation management (#8). Treatment modalities have advanced from traditional fixation (#1) and biological enhancement (#7) to minimally invasive techniques (#6/#9), demonstrating substantially improved outcomes and underscoring the central role of surgical innovation in this field.^[20,21]^ With the growing emphasis on conservative management and rehabilitation, nonsurgical interventions have gained increasing recognition. Studies highlight the critical role of rehabilitation education in the recovery process. For example, goal-oriented dynamic health education for young military personnel has been shown to enhance disease-related knowledge, activities of daily living, and care satisfaction, while reducing postoperative pain and complication rates.^[22]^ A retrospective analysis further reported that nonsurgical treatments achieved short-term outcomes comparable to surgical repair in terms of pain relief, functional improvement, and quality of life, accompanied by lower complication rates.^[23]^

International research trends similarly reflect this transition. The prominence of cluster #1 and keywords such as “low back pain” and “management” signifies growing scholarly attention to symptom control and diversified therapeutic strategies. Predictive modeling suggests that conservative treatment in early-stage lumbar spondylolysis can achieve bony healing rates of up to 90%. Rehabilitation programs incorporating lumbar segmental stabilization training and the resilience and activity combined with training protocol have been shown to effectively alleviate pain and improve functional limitations in patients with spondylolysis and spondylolisthesis.^[24–26]^

A retrospective study of 207 adolescent athletes diagnosed with low back pain and spondylolysis between 2007 and 2019 reported favorable return-to-sport outcomes following a 3-month conservative regimen (including activity modification, thoracolumbar sacral orthosis bracing, and external bone stimulation) supplemented by a 6-week structured rehabilitation program.^[27]^ Another retrospective analysis of 574 pediatric patients with lumbar spondylolysis found that bracing combined with exercise rehabilitation resulted in bony union in 81.7% of cases.^[28]^ Furthermore, a randomized controlled trial demonstrated that foam roller massage, compared to traditional massage, more effectively enhanced dynamic balance recovery, improved lumbar and hip flexibility, and increased lower limb dynamic strength in athletes, suggesting its potential value in sports injury prevention.^[29]^

For the minority of patients who respond poorly to nonsurgical treatment or require surgical intervention, fixation techniques have also advanced. For example, Yang X et al treated 28 young patients with lumbar spondylolysis using a modified single-segment intralaminar screw fixation system combined with autologous cancellous bone grafting, reporting significant pain reduction, functional improvement, and enhanced bone healing.^[30]^

In summary, future research and clinical management of lumbar spondylolysis should prioritize multidisciplinary integration (incorporating expertise from imaging, rehabilitation, and biomechanical engineering) to advance toward precision and individualized treatment paradigms. Conservative treatment and rehabilitation not only play a critical role in symptom management and functional recovery but may also help slow disease progression, decrease the need for surgical intervention, and ultimately improve long-term quality of life.^[31]^

#### 4.2.4. Limitations of the study

This study presents the first systematic and intuitive visualization analysis of the literature and trends concerning lumbar spondylolysis, offering scholars in this field new perspectives for future research. However, our study has several limitations. First, as the literature search was confined to the CNKI and the WOS Core Collection databases, the results may be subject to selection bias. Future studies could benefit from broadening the search scope and conducting more in-depth exploration. Second, the inclusion criteria restricted the language, publication type, and timeframe of the literature, which led to the exclusion of numerous relevant studies and may result in a partial representation of the available data. Third, due to constraints inherent to the CiteSpace software, the analysis of Chinese literature was limited to authors, institutions, and keywords, preventing the acquisition of broader research insights. Finally, the VOSviewer analysis itself has inherent limitations, such as the inability to identify first authors.

## 5. Conclusion

This study utilized CiteSpace and VOSviewer to perform a bibliometric visualization analysis of the literature on lumbar spondylolysis over the past decade. The findings reveal that conservative treatment and rehabilitation interventions have emerged as prominent research hotspots in recent years, reflecting an evolving focus from predominantly surgical management toward integrated, multimodal therapeutic strategies. While the study has certain limitations, primarily related to database selection and search scope, it nevertheless offers a valuable reference for researchers to efficiently identify frontier topics and trends in the field. Future research should prioritize the development of high-quality prospective cohorts, multicenter comparative trials, and multimodal cross-sectional studies. Emphasis should be placed on evaluating the clinical efficacy of nonsurgical interventions combined with structured rehabilitation, thereby strengthening the evidence base for evidence-based clinical decision-making and personalized management approaches.

## Author contributions

**Conceptualization:** Yan Wang^a^.

**Data curation:** Yan Wang^a^.

**Formal analysis:** Yan Wang^a^.

**Funding acquisition:** Chunsheng Qian.

**Investigation:** Chunsheng Qian.

**Methodology:** Yan Wang^c^, Xiaojing Guo.

**Software:** Yan Wang^a^, Chunsheng Qian.

**Supervision:** Yan Wang^c^, Chunsheng Qian.

**Writing – original draft:** Yan Wang^a^, Xiaojing Guo.

**Writing – review & editing:** Yan Wang^c^, Chunsheng Qian.
